# Sensory trick phenomenon improves motor control in pianists with dystonia: prognostic value of glove-effect

**DOI:** 10.3389/fpsyg.2014.01012

**Published:** 2014-09-23

**Authors:** Jakobine Paulig, Hans-Christian Jabusch, Michael Großbach, Laurent Boullet, Eckart Altenmüller

**Affiliations:** ^1^Institute of Music Physiology and Musicians’ Medicine, Hannover University of Music, Drama and MediaHannover, Germany; ^2^Institute of Musicians’ Medicine, Dresden University of Music Carl Maria von WeberDresden, Germany; ^3^Department of Music Physiology, International Piano Academy Lake ComoDongo, Italy

**Keywords:** focal dystonia, sensory trick, glove-effect, botulinum toxin, retraining

## Abstract

Musician’s dystonia (MD) is a task-specific movement disorder that causes loss of voluntary motor control while playing the instrument. A subgroup of patients displays the so-called sensory trick: alteration of somatosensory input, e.g., by wearing a latex glove, may result in short-term improvement of motor control. In this study, the glove-effect in pianists with MD was quantified and its potential association with MD-severity and outcome after treatment was investigated. Thirty affected pianists were included in the study. Music instrument digital interface-based scale analysis was used for assessment of fine motor control. Therapeutic options included botulinum toxin, pedagogical retraining and anticholinergic medication (trihexyphenidyl). 19% of patients showed significant improvement of fine motor control through wearing a glove. After treatment, outcome was significantly better in patients with a significant pre-treatment sensory trick. We conclude that the sensory trick may have a prognostic value for the outcome after treatment in pianists with MD.

## INTRODUCTION

Focal dystonia in musicians, also known as musician’s cramp or musician’s dystonia (MD), is a task-specific movement disorder that manifests itself as a loss of voluntary motor control of extensively trained movements while the musician is playing the instrument (Jankovic and Shale, 1989; Frucht et al., 2001; Hallett, 2006; Altenmüller and Jabusch, 2008). For those who are affected the disorder is highly disabling and often terminates their professional musical careers. MD is still difficult to treat. Therapeutic options mainly include botulinum toxin injections, pedagogical retraining, and anticholinergic medication with trihexyphenidyl (Jabusch et al., 2005).

Pathophysiological theories include deficient inhibition at different levels of the central nervous sensory-motor system (Lin and Hallett, 2009). In hand dystonia, altered somatotopic organization of the putamen was found in a previous study (Delmaire et al., 2005). Furthermore, maladaptive neuronal plasticity leading to a fusion of the digital representations in the somatosensory cortex may play a role. A reduced distance between cortical representational zones of the digits was found in affected musicians (Elbert et al., 1998). In addition to sensory abnormalities, recent findings provide evidence for abnormalities in the somatosensory integration indicating a disturbed interaction between the sensory system and the motor system. A clinical sign, which emphasizes the important role of the sensory system as well as the sensory-motor integration in the pathophysiology of MD is the so-called sensory trick phenomenon. This phenomenon is known from patients with cervical dystonia: touching the face contralaterally or ipsilaterally to the direction of head rotation may reduce or abolish involuntary muscle activity (Wissel et al., 1999; Schramm et al., 2004). In a similar way, patients with blepharospasm (Gómez-Wong et al., 1998) or with oromandibular dystonia (Schramm et al., 2007) may benefit from a contact stimulus in the face. The sensory trick phenomenon has also been described in musicians affected with MD. For example, in pianists with focal hand dystonia playing with a latex glove may lead to a temporary improvement of fine motor control. However, the effect is not stable and only lasts for a few minutes suggesting habituation to altered tactile stimuli (Altenmüller, 2003). Up to now, the specific central nervous mechanisms underlying the sensory trick phenomenon are not fully understood. Using positron emission tomography, successful application of sensory trick stimuli in patients with cervical dystonia resulted in increased activation in the parietal and occipital lobes and decreased activation in the supplementary motor area and the primary sensorimotor cortex (Naumann et al., 2000). Analog activation studies in patients with focal hand dystonia are not yet available.

The sensory trick using a latex glove in musicians with focal hand dystonia as well as its potential association with the outcome after treatment has never been investigated. According to clinical experiences, we hypothesized that patients who presented a strong pre-treatment response to playing with a latex glove in terms of a sensory trick have a better long-term outcome after treatment than those with little initial response. As a rationale for this hypothesis, a strong response to a sensory trick may indicate that the dystonic movement patterns might be easier to modulate and that, as a consequence, treatment might be more effective in these patients. Pianists represent a large fraction of musicians with dystonia (Conti et al., 2008). For assessment of motor control in pianists with MD and for monitoring of treatment effects in these patients, music instrument digital interface (MIDI)-based scale analysis has been reported to be reliable and valid (Jabusch et al., 2004). Using this protocol, the present study was designed to investigate the glove-effect in pianists with dystonia as well as its potential association with the severity of the disorder and with the outcome after treatment. Preliminary results of this research have previously been published (Jabusch et al., 2011).

## PATIENTS AND METHODS

### PATIENTS

Thirty pianists (22 men and eight women; 25 professionals, five amateurs; mean age: 41 ± 13 years, age range: 21–69 years) suffering from MD were included in the study. Details of patients’ characteristics are shown in **Table 1**.

**Table 1:** Patient characteristics.

Case no	Gender	Handedness	Professional/amateur	2. Instrument	Age at start of musical training (years)	Age at diagnosis (years)	Duration of musical training prior to diagnosis (years)
1	M	R	P	organ	7	39	32
2	F	R	P	–	6	24	18
3	M	R	P	–	6	48	42
4	M	R	P	–	7	28	21
5	M	R	P	–	4	25	21
6	F	R	P	–	6	24	18
7	F	L	P	organ	6	29	23
8	F	L	P	–	5	49	44
9	M	R	P	–	6	42	36
10	M	R	P	–	7	45	38
11	M	R	P	–	10	66	56
12	M	R	P	organ	8	41	33
13	M	L	A	–	13	38	25
14	M	R	P	–	8	48	40
15	F	R	P	accordion	8	33	25
16	M	R	P	–	6	52	46
17	F	R	P	–	5	36	31
18	F	both	P	–	11	53	42
19	M	both	A	–	6	50	44
20	M	R	P	–	5	21	16
21	M	R	P	–	8	39	31
22	M	R	P	guitar	9	31	22
23	M	R	P	–	6	25	19
24	M	R	P	organ	9	25	16
25	M	R	P	–	11	53	42
26	M	R	A	–	11	65	54
27	F	R	P	–	7	52	45
28	M	R	P	–	10	25	15
29	M	R	A	–	6	44	38
30	M	R	A	–	8	59	51

Patients were diagnosed at the outpatient clinic of the Institute of Music Physiology and Musicians’ Medicine (IMMM) of the Hannover University of Music, Drama, and Media and underwent complete neurological examination by a neurologist specialized in movement disorders (EA). Patients suffering from additional neurological disorders or from secondary dystonia were excluded from the study. Additional inclusion criteria were: (a) patients were able to play scales at the piano without and with a latex glove; (b) patients were able to come back for a follow-up scale playing test (see Methods) after treatment. Dystonic symptoms manifested themselves in the typical manner as painless cramping of one or more fingers while patients were playing the piano. One pianist suffered from MD in both hands. Data from both hands of this patient were included in the statistical analysis as two separate cases. All participants gave their informed consent to the study which was conducted in accordance with the Declaration of Helsinki.

### METHODS

#### Assessment of motor control

MIDI-based scale analysis was done according to a protocol previously reported (Jabusch et al., 2004). Scales were performed with the affected hand on a digital piano that was connected to a computer. Sequences of 8–15 C major scales were played over two octaves in both playing directions. Scales were played using the conventional C major fingering (1,2,3,1,2,3,4,1,2,3,1,2,3,4,5, and reverse, with the numbers 1–5 referring to the fingers 1–5). The tempo was standardized and paced by a metronome. Desired inter-onset intervals (IOI) were 125 ms in 26 patients. Due to severely impaired motor control in four patients, desired IOI were 187.5 ms in three patients and 375 ms in one patient. The temporal unevenness of IOI has previously been identified as a valid, reliable and precise indicator of the impairment of motor control in pianists with dystonia (Jabusch et al., 2004). For each participant temporal unevenness of IOI was analyzed for the affected hand and for each playing direction by calculating the median of the standard deviations of IOI (medSD-IOI) of all scales, respectively. The more severely affected playing direction was identified by the higher medSD-IOI score. This measure was used for all analyses.

Performance tests were carried out separately under three conditions: (a) baseline test before treatment (termed “baseline”); (b) playing with latex glove (sensory trick) before treatment (termed “glove”); (c) follow-up test after treatment (termed “follow-up”). The glove test was performed 10 min after the baseline test. For the test, a latex glove was selected that comfortably fit the patient’s hand size (Proline Nitril®: small, medium, or large). Besides the glove test, no other attempts were made to provoke a sensory trick phenomenon.

#### Treatment strategies

Between the first and the last visit at the IMMM, the patients were participating in an open-label trial undergoing therapies recommended by the treating physicians or self-selected by the musicians. Therapeutic approaches, as monotherapies or in simultaneous or successive combination, included the following options: botulinum toxin injections (BTX) were applied in patients in which primary dystonic movements could be clearly distinguished from secondary compensatory movements. Target muscles were identified by visual inspection of the dystonic movement patterns while patients were playing their instruments. A lyophilized BTX A powder (Dysport®, Ipsen Ltd., Berkshire, UK) was injected using an EMG-guided technique (Karp et al., 1994). Pedagogical retraining (PR) was taking place under the supervision of a piano instructor (author Laurent Boullet) specialized in dystonia retraining. PR included elements based on the following principles reported previously (Boullet, 2003): (1) movements of affected body parts were limited to a level of tempo and force at which the dystonic movement would not occur; (2) compensatory movements (e.g., of adjacent fingers) were avoided, partially under the application of splints; (3) instant visual feedback with mirrors or monitors helped patients to recognize dystonic and non-dystonic movements. Trihexyphenidyl (TRHX) was frequently applied in addition to BTX or PR if no contraindication was present. Adjustment of the dosage was made depending on beneficial effects and side effects.

#### Assessment of patients’ history and treatment details

Information on symptoms (e.g., age at onset) and pharmacological treatment was yielded by means of a questionnaire and retrospective chart reviews. Information on retraining therapy (e.g., number of sessions, intervals between sessions) was documented and provided by the supervising piano instructor.

### STATISTICAL ANALYSIS

Potential associations between variables were assessed using Spearman correlations, for normally distributed variables we used Pearson correlations. On the individual level, glove-effect and outcome of patients were analyzed by Mann–Whitney *U* tests using medSD-IOI values of the baseline, glove or follow-up tests. Between-group differences were analyzed by Mann–Whitney *U* tests and Fisher’s exact tests, within-group differences were assessed by Wilcoxon signed-rank tests. All statistical tests were two-tailed if not stated otherwise. The alpha level was set at 0.05.

## RESULTS

The mean age at onset of dystonic symptoms was 33.5 years (range, 14–58); median duration of symptoms at the time of the baseline and glove tests was 2.7 years, (0.2–44.6). Follow-up tests were conducted on average 4.8 ± 2.5 years after baseline tests. Scale analysis tests revealed following results: median medSD-IOI_baseline_ was 18.9 ms (range, 10.4–35.6); median medSD-IOI_glove_ was 20.2 ms (10.3–48.2); median medSD-IOI_follow-up_ was 20.0 ms (10.4–30.2). In the mentioned four cases with increased target-IOI, adjustment factors between 0.52 and 0.89 were applied that were obtained from a group of 14 healthy pianists for medSD-IOI results of scales played in different tempi (unpublished data).

### GLOVE-EFFECT

**FIGURE 1:**
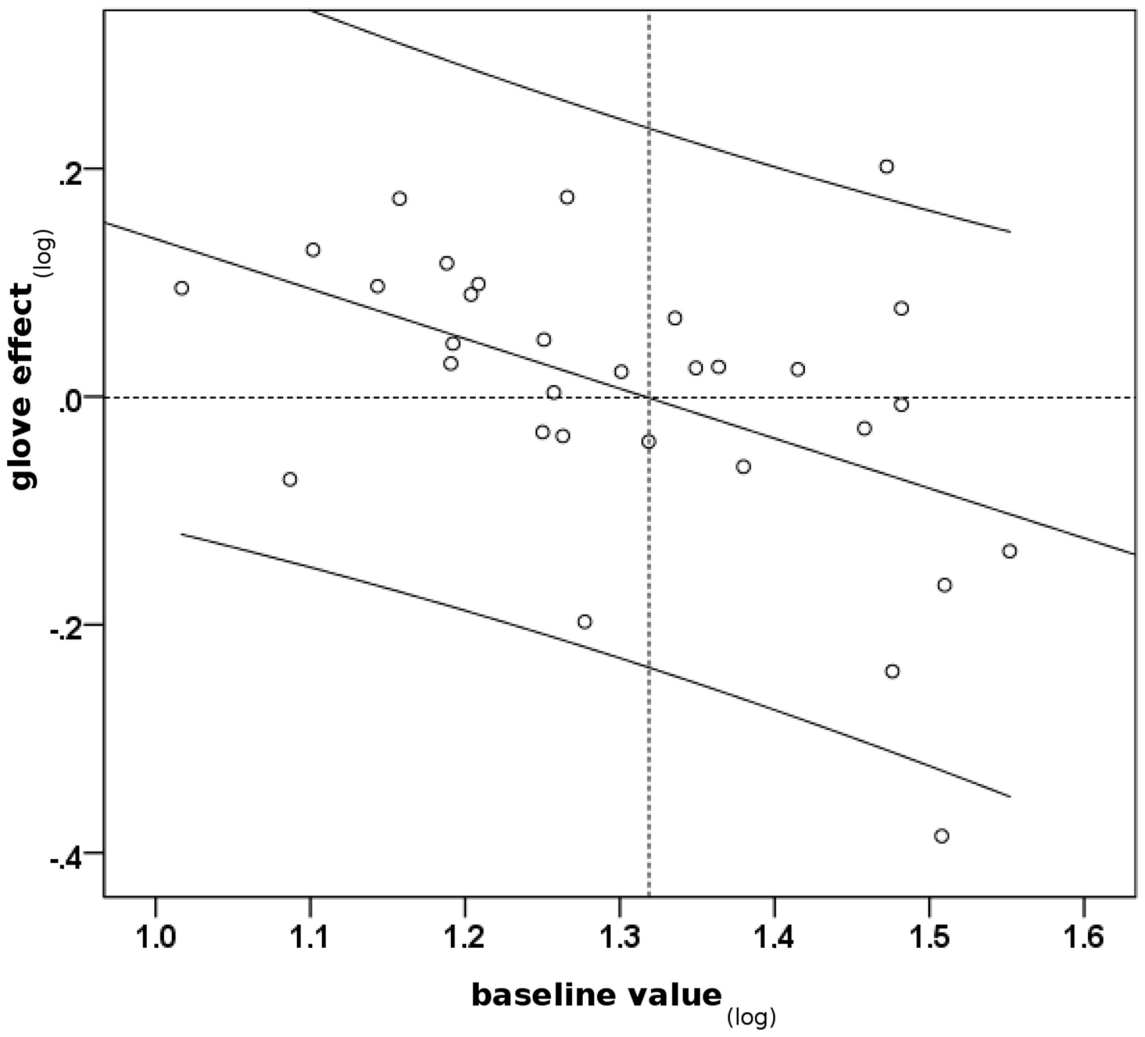
**Scatterplot indicating an association between severity of dystonia at the time of baseline test and change of performance through the glove-effect.** Baseline value_(log)_: log medSD-IOI_baseline_, high values indicate severely impaired motor control. Glove-effect_(log)_: log medSD-IOI_glove_ – log medSD-IOI_baseline_, negative values indicate improvement through glove and vice versa. Pearson’s *r* = -0.45; *p* = 0.01, two-tailed. Black line: regression line. Curved lines: 95% confidence interval.

The comparison between the performance at baseline and that in the glove-test was assessed for each patient individually. Mann-Whitney *U* tests showed significant improvement of fine motor control through wearing a glove in six cases in comparison with the baseline (19%; beneficial glove-effect; BGE; all *p*-values < 0.05), significant aggravation through the glove in nine cases (29%; all *p*-values < 0.05) and no significant effect in 16 cases (52%; both groups considered as displaying no beneficial glove-effect; no BGE). The glove-effect (GE) was described as (medSD-IOI_glove_–medSD-IOI_baseline_). Median GE was 1.3 ms (range -17.7, 17.9), negative values indicating improved motor control with the glove compared to baseline. The severity of MD at the time of baseline correlated with GE (Pearson’s *r* = -0.38; *p* = 0.03): the more pronounced dystonic symptoms were, the higher was the improvement with the glove. As a consequence of the asymptotic properties of performance values in the expertise-performance relationship, GE was logarithmically rescaled according to the following equation: GE_log_ = log medSD-IOI_glove_–log medSD-IOI_baseline_. GE_log_ correlated with log medSD-IOI_baseline_ (Pearson’s *r* = -0.45; *p* = 0.01; see **Figure 1**).

### LONG-TERM OUTCOME

On an individual level, Mann-Whitney *U* tests revealed significant improvement of MD symptoms in 12 cases (39%; all *p*-values < 0.05), significant deterioration in seven cases (22%; all *p*-values < 0.05) and no significant change in 12 cases (39%).

### TREATMENT STRATEGIES

Eight patients (27%) were consequently treated with BTX (consequent treatment was defined as minimum three BTX injections). BTX treatment details were as follows: median treatment duration 28.7 months (range, 5–70); median number of injections 4 (3–19); median interval between injections 5.3 months (3–23); median minimum dosage 45 units (5–200); median of the median dosage 65 units (33–360); median maximum dosage 100 units (50–500). 21 patients (70%) consequently took part in PR (defined as minimum 4 months PR). PR treatment details were as follows: median treatment duration 34.7 months (4–77); median number of PR sessions 33 (3–133); median interval between sessions 1 month (0.5–2.3). Seven patients (23%) were consequently treated with TRHX (defined as minimum 4 months TRHX application). TRHX treatment details were as follows: median treatment duration 16 months (5–31); median maximum dosage 8 mg/day (2–15); median best dosage 6 mg/day (1.25–10). Treatment strategies were either applied as monotherapy (BTX: *n* = 3; PR: *n* = 13; TRHX: *n* = 1), as dual therapy (PR/BTX: *n* = 2; PR/TRHX: *n* = 3) or as triple therapy (BTX/PR/TRHX: *n* = 3). Five patients treated with PR and/or BTX and/or TRHX did not meet the aforementioned criteria for “consequent” treatment.

Outcome was defined as medSD-IOI_follow-up_–medSD-IOI_baseline_(outcome value; OV). Median OV was -2.0 ms (-14.2; 12.4). Analogously to GE_log_, OV_log_ was defined as = log medSD-IOI_follow-up_–log medSD-IOI_baseline_. There was a correlation between GE_log_ and OV_log_ (Pearson’s *r* = 0.56, one-tailed *p* = 0.03) for patients with GE < 0. This correlation indicates that in the group of patients with a GE < 0, i.e., for those patients with no initial deterioration through the glove, an association was observed between the glove-effect and the outcome: patients with better improvement by wearing the glove had a better outcome.

**FIGURE 2:**
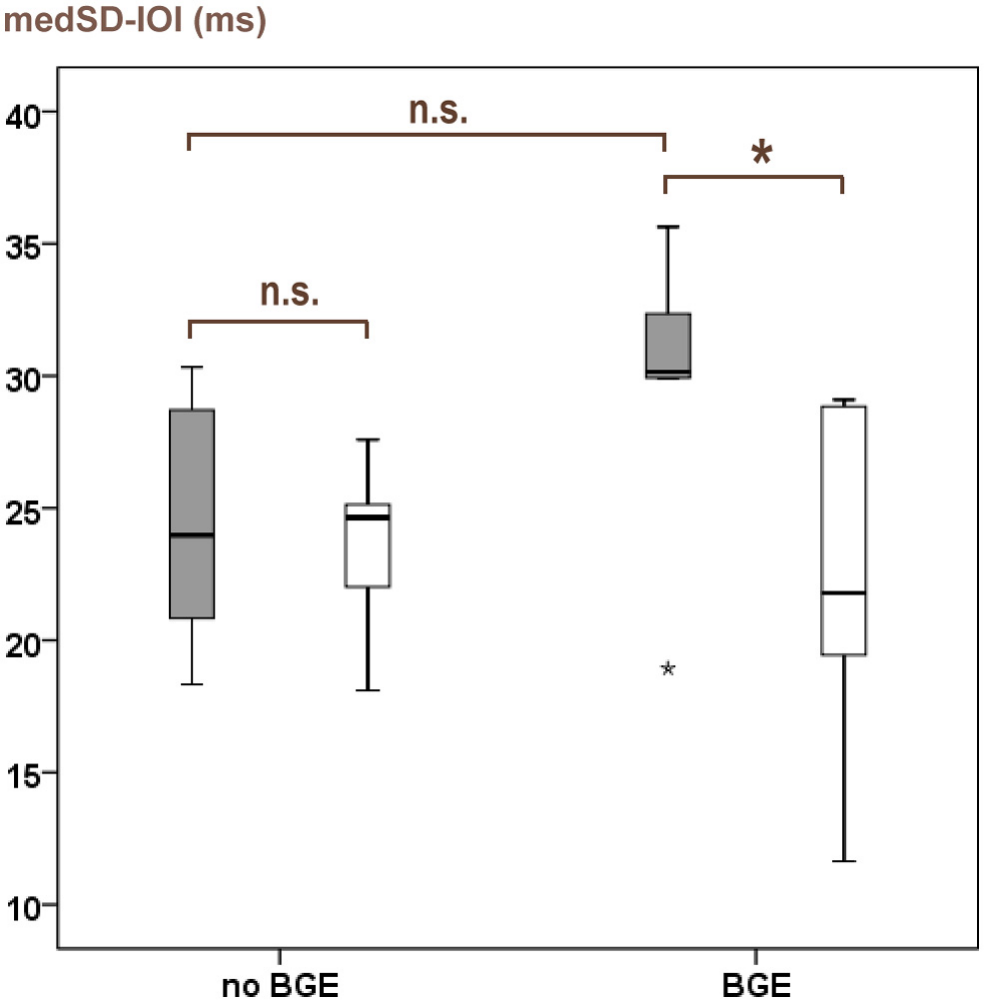
**Outcome results of consequently treated patients with no initial deterioration through the glove (glove effect < 0): comparison between patients with a beneficial glove-effect (BGE) and those with no BGE.** Gray bars: performance values at baseline (medSD-IOI_baseline_). Light bars: performance values at follow-up (medSD-IOI_follow-up_). High medSD-IOI values indicate poor motor control and vice versa. *p < 0.05 (one-tailed Wilcoxon test); n.s., not significant.

All further statistical analyses were made on those patients with a GE < 0 (patients with no initial deterioration through the glove) who were consequently treated as stated above. This group included five patients with a BGE and five patients with no BGE. Median OV was -7.3 ms (range -10.7, -1.1) in patients with BGE and -2.7 ms (-5.2, 9.2) in patients with no BGE. Both groups did not differ in medSD-IOI_baseline_ values (Mann–Whitney *U* test; two-tailed *p* = 0.2). In patients with BGE, medSD-IOI_follow-up_ values were lower than medSD-IOI_baseline_ values (Wilcoxon test; one-tailed *p* = 0.03; see **Figure 2**) indicating that motor control significantly improved after treatment. This was not the case in patients with no BGE (Wilcoxon test; one-tailed *p* = 0.3). Patients with a BGE showed significant better OVs compared to the patients with no BGE (Mann–Whitney *U* test; one-tailed *p* = 0.048). Fisher’s exact tests revealed that patients with a BGE did neither receive different treatment regimes than patients with no BGE with respect to BTX and PR (*p*-values > 0.05). Mann–Whitney *U* tests for therapy details revealed that patients with a BGE did neither differ in the duration of follow-up nor in treatment details from patients with no BGE: in BTX patients no difference was found in treatment-duration, number of injections and dosages; PR patients did not differ in duration of treatment, number and interval of sessions (all *p*-values > 0.05).

## DISCUSSION

The present study investigated the sensory trick phenomenon using a latex glove in pianists with focal hand dystonia as well as its potential association with the outcome after treatment. Playing with the glove, six patients (19%) showed a statistically significant improvement of motor control compared to baseline. This finding is in agreement with other publications (Gómez-Wong et al., 1998; Schramm et al., 2004, 2007) reporting that only a minority of patients with focal dystonia exhibited a reduction of dystonic muscle contractions by using a sensory trick maneuver.

### ASSOCIATION BETWEEN GLOVE-EFFECT AND SEVERITY OF MD

According to the findings, the potential benefit of playing with a latex glove was associated with the severity of dystonic symptoms. The correlation between baseline motor control assessed by MIDI-based scale analysis (log medSD-IOI_baseline_) and the glove-effect (GE_log_) indicated that pianists with more severely pronounced symptoms of dystonia showed a better sensory trick benefit of playing with a glove. In contrast, patients with less pronounced symptoms of dystonia had a smaller benefit or even a deterioration of their performance while playing with the glove. An explanation for this finding may be the mechanical constraints pianists suffer from playing with a glove that limits their finger movements to a certain extent. Obviously, the glove is able to cause two competing effects: (a) an improvement of dystonic symptoms in terms of a sensory trick phenomenon and (b) a constraint of pianistic movements through mechanical impediment. According to the scatter plot in **Figure 1**, the log medSD-IOI_baseline_ cut-off value between both effects is approximately 1.32 which corresponds to a medSD-IOI_baseline_ value of approximately 21 ms. Below this value motor control of pianists with dystonia is relatively well-preserved with the consequence that (a) mechanical constraints by the latex glove have a negative effect on playing ability and (b) the response of subtle dystonic symptoms to the glove in terms of a sensory trick phenomenon may be limited due to a ceiling effect. Beyond the cut-off value of 21 ms, due to the severity of dystonic symptoms, mechanical constraints through the glove were less prominent whereas the positive influence of the sensory trick prevailed.

### LONG-TERM OUTCOME AND ITS ASSOCIATION WITH THE GLOVE-EFFECT

After an average follow-up time of 4.8 years 39% of patients showed a statistically significant improvement of motor control compared to baseline whereas 39% displayed no change of dystonic symptoms and 22% demonstrated a significant deterioration. In a recent publication, objective gains in task-specific motor performance were documented in 42.9% of pianists with MD after treatment and after similar follow-up periods (van Vugt et al., 2014). The similarity of results is partially explained with a substantial overlap of the present patient group with the patients reported in that publication. A previous study (Jabusch et al., 2009) investigating the long-term outcome of MD patients revealed a higher percentage rate of patients with an improvement in 71% using the same measurement methods. The following reasons may be responsible for this observation: (a) different samples with only partial overlap were investigated in both studies; (b) sample sizes were relatively small in both studies.

According to the findings, initial improvement of motor control with a latex glove was associated with long-term improvement after therapy. The correlation between the glove-effect (GE_log_) and the improvement in the follow-up test (OV_log_) in patients with GE <0, i.e., for those patients with no initial deterioration through the glove, indicated that pianists with a strong initial glove-induced benefit had a better treatment response than those with less benefit through the glove. In the subgroup of consequently treated patients, long-term OVs of pianists with a beneficial glove-effect (BGE) were significantly better compared to those without a BGE. The outcome differences could not be explained with the abovementioned worse baseline performance in patients with stronger glove-response because these two subgroups did not significantly differ in baseline performance values. Outcome differences could also not be explained by treatment strategies because both groups displayed no significant differences in treatment regimens, treatment durations or other treatment details. Therefore, findings strengthen the clinical impression that the glove may act as a predictor for the clinical development of patients with MD and that a BGE may have a prognostic value in the treatment of MD: in patients with no detrimental glove response, a significant pre-treatment improvement with the glove seems to predict a better long-term outcome after treatment than a small or no initial improvement. Positive outcome of the six patients with a BGE was reflected by their musical situation at the end of the follow-up period: five pianists were able to proceed with the activities that they had pursued before onset of dystonia [as chamber musician (1), repetiteur (1), piano teacher (1), amateur pianists (2)]. One pianist of this group had switched from playing chamber music to piano teaching.

The pathophysiological background for the association between the sensory trick and treatment outcome remains unclear. We speculate that a strong sensory trick response may indicate that dystonic movement patterns are easier to modulate and that therefore treatment was more effective in these patients. Further research has to be conducted to clarify the question if pathophysiological alterations in patients with MD such as findings of maladaptive plasticity are less pronounced in patients with a stronger sensory trick response. An analogy may exist between the sensory trick phenomenon and the so-called external focus in motor learning that has been reported to enhance motor skill learning in sports science (Wulf and Prinz, 2001). From this perspective, improvement of dystonic symptoms through the sensory trick might partially be explained by an externalization of the attentional focus by wearing the glove.

### STRENGTHS AND LIMITATIONS OF THE STUDY

Strengths of this study include the use of an objective, validated measure of motor performance to quantify the sensory trick in a relevant musical task. Another strength is the inclusion of a very homogeneous patient group with respect to the task affected by MD. An important limitation is the relatively small number of patients, especially of those who displayed an improvement through the glove. As a consequence, it can not be completely excluded that the better outcome of patients with a better glove response may at least partially be a consequence of the fact that strongest glove effects were related to most severe dystonia which gives room for more improvement after treatment. This question has to be addressed in future studies in larger patient groups with homogeneous baseline performances and different glove responses. At present, the inclusion of non-keyboard musicians with the aim to enlarge the study group was, however, not possible due to the fact that objective and validated assessment of motor control and its impairment in MD is available only for pianists so far.

## CONCLUSION

In affected pianists with no pre-treatment deterioration of motor control through the glove, the sensory trick may have a prognostic value for the outcome after treatment in pianists with MD.

Until now, the sensory trick has not successfully been included in the rehabilitation of musicians with MD due to habituation. Future attempts should clarify if variable modulation of the sensory input, e.g., by variable tactile stimuli, with the aim of a prolonged external focus and reduced habituation may enable the applicability of the sensory trick in rehabilitation of musicians with MD.

## DOCUMENTATION OF AUTHOR ROLES

(1) Research project: (A) Conceptions, (B) Organization, (C) Execution
(2) Statistical Analysis: (A) Design, (B) Execution, (C) Review and Critique
(3) Manuscript: (A) Writing of the first draft, (B) Review and Critique
Jakobine Paulig: 1A, 1B, 1C, 2A, 2B, 2C, 3A, 3B
Hans-Christian Jabusch: 1A, 1C, 2A, 2B, 2C, 3B
Michael Großbach: 1A, 2A, 2B, 2C
Laurent Boullet: 1C, 2C, 3B
Eckart Altenmüller: 1A, 1B, 2C, 3B

## Conflict of Interest Statement

The authors declare that the research was conducted in the absence of any commercial or financial relationships that could be construed as a potential conflict of interest.
